# Calciprotein particles and fibroblast growth factor 23 contribute to the pathophysiology of hypercalcemia in a patient with renal sarcoidosis

**DOI:** 10.1093/ckj/sfz086

**Published:** 2019-08-04

**Authors:** Yoshitaka Iwazu, Makoto Kuro-o, Yutaka Miura, Shin-ichi Takeda, Toshiyuki Yamada, Daisuke Nagata

**Affiliations:** 1 Department of Nephrology, Jichi Medical University, Shimotsuke, Tochigi, Japan; 2 Department of Clinical Laboratory Medicine, Tochigi Medical Center Shimotsuga, Jichi Medical University, Shimotsuke, Tochigi, Japan; 3 Department of Medicine, Division of Nephrology, Jichi Medical University, Shimotsuke, Tochigi, Japan; 4 Division of Anti-aging Medicine, Center for Molecular Medicine, Jichi Medical University, Shimotsuke, Tochigi, Japan

**Keywords:** bisphosphonate, nephrocalcinosis, nephrolithiasis, phosphatemia, vitamin D

## Abstract

In patients with sarcoidosis, dysregulated calcium metabolism is one of the frequently observed complications. However, little attention has been paid to abnormal phosphate metabolism. Herein we present the case of a 42-year-old Japanese man with renal sarcoidosis who developed acute kidney injury due to hypercalcemia and nephrolithiasis. Laboratory data showed hypercalcemia with a normal serum phosphate level and high serum 1,25-hydroxyvitamin D_3_, fibroblast growth factor 23 (FGF23) and calciprotein particle (CPP) levels. After treatment with oral prednisone and bisphosphonate, the laboratory abnormalities and renal dysfunction were resolved. Thus increases in FGF23 and CPP may indicate disturbed phosphate metabolism in renal sarcoidosis.

## INTRODUCTION

Sarcoidosis is characterized by the presence of noncaseating granulomas that synthesize 1α-hydroxylase, which converts the inactive form of vitamin D (25-hydroxyvitamin D_3_) into the active form of 1,25-dihydroxyvitamin D_3_ [1,25(OH)_2_D_3_]. Thus sarcoidosis patients have high serum levels of 1,25(OH)_2_D_3_, which lowers serum parathyroid hormone (PTH) levels [1]. Because of the high 1,25(OH)_2_D_3_ and low PTH, serum phosphate levels would be expected to increase in sarcoidosis patients. However, hyperphosphatemia is observed in only 6% of these patients, whereas the prevalence of hypercalcemia is 18%. Moreover, hypophosphatemia is observed in 7% of patients with sarcoidosis and no association has been demonstrated between abnormal phosphate levels and dysregulated calcium metabolism [2]. These findings suggest that unknown mechanisms might cause impaired phosphate homeostasis. Herein we report an interesting case of renal sarcoidosis that may provide a clue to explore the mechanism responsible.

## CASE REPORT

A 42-year-old Japanese man was diagnosed with acute kidney injury secondary to bilateral obstructive ureteric calculi and underwent emergency bilateral retrograde ureteric stenting in our center [serum creatinine (SCr) 12.71 mg/dL] in March 2014. After stenting, the renal function improved significantly (SCr 1.49 mg/dL). Six weeks later, extracorporeal shock wave lithotripsy was performed and both stents were removed. Stone analysis revealed it to be 30% calcium phosphate and 70% calcium oxalate. Four months later he was admitted to our unit because his SCr had not improved any further.

The laboratory tests were notable for the following serum levels: creatinine 1.82 mg/dL, calcium 12.0 mg/dL, phosphate 3.4 mg/dL, calciprotein particle (CPP) 191 040 AU (measured using the OsteoSense method [3]), albumin 4.5 g/dL, intact PTH 8 pg/mL (reference range 10–65), PTH-related protein <1.1 pmol/L (reference range <1.1), 1,25(OH)_2_D_3_ 112 pg/mL (reference range 20–60), intact fibroblast growth factor 23 (FGF23) 2925 pg/mL (reference range 16–69), angiotensin-converting enzyme 30.0 mU/mL (reference range 7.7–29.4) and lysozyme 36.1 μg/mL (reference range 5–10.2). Urine studies showed no dipstick proteinuria, no hematuria, mild pyuria and no casts. Fractional excretion of calcium and phosphate were 14 and 38%, respectively. Noncontrast computed tomography revealed many bilateral kidney stones, renal calcifications and multiple ill-defined nodules in the bilateral lungs, with bilateral hilar and mediastinal lymphadenopathies. There was a history of uveitis 5 years prior.

**FIGURE 1:**
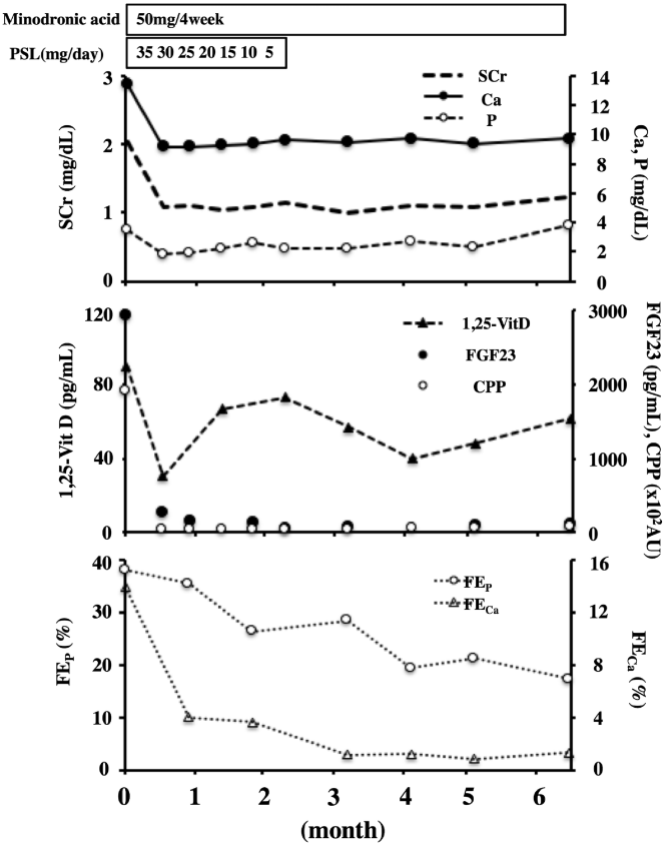
Clinical course of SCr, calcium (Ca), phosphate (P), 1,25(OH)_2_D_3_ (1,25-VitD), FGF23, CPPs and fractional excretion of calcium (FE_Ca_) and phosphate (FE_P_).

The patient declined to undergo renal biopsy. Therefore, based on the clinical and laboratory findings, he was diagnosed as having sarcoidosis complicated by hypercalcemia. Prednisolone (PSL) (35 mg/day, 0.4 mg/kg) and bisphosphonate (minodronic acid 50 mg/4 weeks) were started, which immediately led to a decrease in the serum levels of calcium, SCr, CPP, 1,25(OH)_2_D_3_ and FGF23 (Figure 1). The prednisolone was gradually tapered off and weaned 6 months later. At 52 months follow-up, there has been no relapse.

## DISCUSSION

To our knowledge, this is the first report to show that serum FGF23 and CPP levels were increased in hypercalcemia due to sarcoidosis. FGF23 functions as a phosphaturic hormone and maintains the phosphate balance [4]. CPPs are nanoparticles composed of serum protein fetuin-A and solid-phase calcium phosphate and are dispersed in the blood. CPP levels increase following increases in serum phosphate and the calcium phosphate product [5]. Therefore the increase in FGF23 and CPP in this sarcoidosis patient may indicate disturbed phosphate metabolism and may be a result of hypercalcemia caused by excess 1,25(OH)_2_D_3_ synthesis.

In acute sarcoidosis patients with normal kidney function, 15.4% of patients had detectable levels of serum FGF23 and 50% had FGF23 levels above the upper limit of normal [5]. In that particular study, serum FGF23 levels were measured and associated with higher serum calcium and lower serum intact PTH levels, but serum 1,25(OH)_2_D_3_ levels were not measured [5]. In our case, the serum FGF23 level before treatment was obviously higher than that of other patients with comparable estimated glomerular filtration rates [3]. Our case suggested that the increase in FGF23 might compensate for increased dietary absorption of phosphate due to the high 1,25(OH)_2_D_3_, and reduced urinary excretion of phosphate due to low PTH and low renal Klotho expression, contributing to maintaining the normal serum phosphate levels.

Because blood is supersaturated in terms of calcium and phosphate ions, even a slight, transient increase in calcium and/or phosphate concentrations can trigger the precipitation of calcium phosphate and the formation of CPP [4]. In fact, the serum CPP levels in this case decreased significantly as the serum calcium and FGF23 levels decreased after treatment (Figure 1). Therefore increased serum CPP levels might reflect disturbed phosphate metabolism in addition to disturbed calcium metabolism. Considering the fact that serum CPP levels correlate with both serum calcium and serum FGF23 levels but not serum 1,25(OH)_2_D_3_ levels (Figure 1), it is intriguing to speculate that CPP may stimulate the secretion and/or production of FGF23.

We did not investigate renal Klotho expression. However, the clinical course demonstrating that the levels of fractional excretion of phosphate remained high, despite the marked decrease in serum FGF23 levels at 1 month after treatment, suggests indirectly that renal Klotho expression has decreased before treatment (Figure 1). The serum FGF23 level before treatment was also higher than that of patients in chronic kidney disease Stage 5 [3] with extremely low renal Klotho expression. Therefore the decrease in renal Klotho expression could have contributed to the increase in serum FGF23 level, at least in part.

In renal sarcoidosis, hypercalcemia and increased serum CPP levels may promote the production of FGF23, which may result in suppression of hyperphosphatemia that could otherwise have been caused by increased 1,25(OH)_2_D_3_. We propose that CPP and FGF23 may contribute to the pathophysiology of renal sarcoidosis.

## FUNDING

This work was supported in part by Japan Society for the Promotion of Science KAKENHI Grant JP16K08941.

## CONFLICT OF INTEREST STATEMENT

None declared.

## References

[1] Hilderson I , Van Laecke S, Wauters A et al Treatment of renal sarcoidosis: is there a guideline? Overview of the different treatment options. Nephrol Dial Transplant 2014; 29: 1841–1847 24235078 10.1093/ndt/gft442

[2] Lim V , Clarke BL. Coexisting primary hyperparathyroidism and sarcoidosis cause increased angiotensin-converting enzyme and decreased parathyroid hormone and phosphate levels. J Clin Endocrinol Metab 2013; 98: 1939–1945 23493435 10.1210/jc.2012-4197

[3] Miura Y , Iwazu Y, Shiizaki K et al Identification and quantification of plasma calciprotein particles with distinct physical properties in patients with chronic kidney disease. Sci Rep 2018; 8: 1256 29352150 10.1038/s41598-018-19677-4 PMC5775250

[4] Kuro-o M. The Klotho proteins in health and disease. Nat Rev Nephrol 2019; 15: 27–44 30455427 10.1038/s41581-018-0078-3

[5] Sexton DJ , O'Reilly MW, Geoghegan P. Serum fibroblastic growth factor 23 in acute sarcoidosis and normal kidney function. Sarcoidosis Vasc Diffuse Lung Dis 2016; 33: 139–142 27537716

